# Evaluation of the Effect of Vitamin D Levels During the Last Trimester of Pregnancy on Fetomaternal Outcomes in Patients With Preeclampsia

**DOI:** 10.7759/cureus.49145

**Published:** 2023-11-20

**Authors:** Shweta Saini, Kalpana Kumari, Priyanka Rai, Taranpreet Kaur, Manish Raj

**Affiliations:** 1 Obstetrics and Gynaecology, Uttar Pradesh University of Medical Sciences, Etawah, IND; 2 Obstetrics and Gynaecology, All India Institute of Medical Sciences, Deoghar, Deoghar, IND; 3 Orthopaedics, All India Institute of Medical Sciences, Deoghar, Deoghar, IND

**Keywords:** vitamin d, preeclampsia, multisystem disorders, fetomaternal outcome, hypertension

## Abstract

Introduction

Preeclampsia is a multisystem disorder with hypertension after 20 weeks of gestation. Among many predictors of preeclampsia, vitamin D being one of them is under many studies for establishing a correlation between levels of vitamin D and preeclampsia.

Objective

To observe a relation between vitamin D levels and preeclampsia and assess related fetomaternal outcomes.

Method

It is an observational study at the tertiary care center. One hundred twenty patients, out of which 60 were taken as cases with BP>140/90, and 60 were taken as controls with normal BP in a tertiary care center from January 1, 2020, to June 30, 2021. All investigations were sent, and the mode of delivery and the fetomaternal outcome were assessed.

Results

Compared to normal pregnant patients, preeclamptic patients have significantly lower levels of vitamin D with a p-value of <0.001, which is significant.

Conclusion

There is a relationship between vitamin D levels and preeclampsia. However, the effects of supplementation of vitamin D on fetomaternal outcomes need further studies.

## Introduction

There is a wide spectrum of the underlying reasons for hypertension in pregnancy. It is typically diagnosed when the systolic blood pressure (BP) reaches or exceeds 140 mmHg and/or the diastolic pressure reaches or exceeds 90 mmHg. To establish a diagnosis, these measurements must be obtained on two separate occasions at least six hours apart, within a week. Alternatively, a single reading of diastolic BP exceeding 110 mmHg in pregnant women is also considered indicative of hypertension for the purposes of evaluation and management. Hypertensive disorders during pregnancy are categorized into three types: gestational hypertension, preeclampsia and eclampsia, and chronic hypertension. The most common are preeclampsia and eclampsia [1].

Preeclampsia is a multisystem disorder characterized by hypertension after 20 weeks of gestation that complicates nearly 2%-8% of pregnancies; it affects both mother and fetus and increases both maternal and fetal morbidity and mortality [2-4]. There are two types of preeclampsia: severe and non-severe. Its severity is indicated by systolic BP >160 mmHg, diastolic BP >110 mmHg, presence of proteinuria, occurrence of headache and visual disturbances, upper abdominal pain, and any sign of end-organ damage. Symptoms of end-organ damage include oliguria (<500 mL/24 hours), elevated serum transaminases, and fetal growth restriction [5]. Although the exact etiopathogenesis remains unknown, some well-known etiologies include abnormal placentation, endothelial dysfunction and vasospasm, genetic factors, pre-angiogenic and anti-angiogenic factors, immunologic tolerance, and oxidative stress. Several predictors of preeclampsia must also be considered; these are human chorionic gonadotropin (hCG), alfa-fetoprotein (AFP), estriol, pregnancy-associated plasma protein A (PAPP A), inhibin A, activin A, kisspeptin, C-reactive protein, cytokines, placental growth factor, vascular endothelial growth factor (VEGF), FMS-like tyrosine kinase receptor-1 (sFlt-1), endoglin, antithrombin-3, atrial natriuretic peptide (ANP), and 25-hydroxyvitamin D (vit D3) [5].

Vitamin D3 is also known as cholecalciferol. Classically, it regulates calcium hemostasis, bone health, and neuromuscular functions, but recent advances and studies have revealed that it regulates ~200 human genes, resulting in different autocrine effects in different tissues [6]. Moreover, calcitriol modulates immune function through a rapid action pathway by binding to receptors on the plasma membrane when the active hormone is synthesized in situ by several extrarenal tissues, such as macrophages, endothelium, and keratinocytes [7]. Furthermore, processes such as cell proliferation, differentiation, and apoptosis involve vitamin D [7]. Immune responses that regulate both innate and adaptive immunity are also regulated by vitamin D [8]. Additionally, vitamin D deficiency is commonly associated with hypertensive disorders, which are the most prevalent complications of pregnancy.

## Materials and methods

This study was conducted on 128 antenatal patients admitted to the emergency labor room of the obstetrics and gynecology department of our hospital, from January 1, 2020, to June 30, 2021. It was a hospital-based observational study conducted in a tertiary care center. After obtaining ethical clearance from the Institute's ethical committee, all the women fulfilling inclusion criteria of age 18-40 years after 37 weeks of gestation were included, and written and informed consent was obtained from all the study participants. Subjects were divided into two groups, 64 antenatal patients in each group were taken as follows:

Group A: Patients with preeclampsia

Group B: Patients with normal blood pressure

Blood samples were collected from all the participants and were sent for the evaluation of vitamin D and others. Reports were collected, and a comparison of Vitamin D levels and their fetomaternal outcome of patients with preeclampsia with normal pregnancy was done.

Patients with chronic illness, severe cardiovascular disease or failure, liver/renal disease, congenitally malformed baby/IUD, twin pregnancies, known cases of HTN and DM, and eclampsia were excluded. A detailed history from the patients regarding parity, age, gestational age, socioeconomic status, booking history, history of iron and calcium intake, and history suggestive of imminent symptoms, personal, past, and family history was taken to exclude the abovementioned exclusion criteria. Obstetrical history was taken with special reference to any complications, such as recurrent abortions, intrauterine death, IUGR, preeclampsia, and multiple pregnancies in previous pregnancies. A thorough general examination and obstetric examination were performed with special attention to pallor, icterus, edema, blood pressure, and weight of the patient at the beginning of the study. Systemic examination of the cardiovascular system, central nervous system, and respiratory system was done to exclude the presence of any systemic disease. Assessment of fetal well-being was conducted by clinical (fetal heart rate) and ultrasound evaluation. All necessary investigations such as CBC, LFT/KFT, LDH, S. uric acid, PTI/INR, BT/CT, etc., and follow-up were done for a fetomaternal outcome like mode of delivery, APGAR score, and NICU admission. Data were compiled using MS Excel and analyzed using Statistical Product and Service Solutions (SPSS, version 20) (IBM SPSS Statistics for Windows, Armonk, NY), and a p-value of <0.05 was considered statistically significant. To compare the levels of vitamin D in both groups. As the data are not normally distributed, the Mann-Whitney U test is used for the comparison of vitamin D levels in both of the groups. To compare the fetomaternal outcome in both groups, a chi-square test is used.

## Results

A total of 128 patients were recruited for this observational study and divided into two groups: Group A (those with preeclampsia) and Group B (those with normotensive antenatal hypertension). This study aimed to determine vitamin D levels in preeclampsia patients and compare their results and their fetomaternal outcomes to normal pregnancy.

Table 1 shows the characteristics of the patients. The majority of those in Group A fall within the age range of 18-23 years (64.1%), while a significant proportion of patients in Group B are in the age range of 24-28 years (45.3%); with a p-value of 0.027, the difference between the two groups was considered statistically significant. Moreover, the BMI of most patients in Group A is 25-29.9 (56.2%), whereas the BMI of the majority of those in Group B is 18.5-24.9 (75%). Furthermore, in Group A, most of the patients were primigravida (57.8%), while the remaining were multigravida (42.2%). In Group B, a smaller proportion of the patients were primigravida (35.9%), while the majority were multigravida (64.1%); the difference between the two groups was found to be statistically significant, with a p-value of <0.05.

**Table 1:** Showing Demographics of Patients

Age	Group A	Group B	P-value
18–23 yrs	41 (64.1%)	25 (39.1%)	0.027
24–28 yrs	14 (21.9%)	29 (45.3%)	
29–33 yrs	6 (9.4%)	7 (10.9%)	
>34 yrs	3 (4.6%)	3 (4.6%)	
BMI			<0.001
<18.5	0	2 (3.1%)	
18.5–24.9	17 (26.6%)	48 (75%)	
25–29.9	36 (56.2%)	11 (17.2%)	
>30	11 (17.2%)	3 (4.7%)	
Parity			0.013
Primigravida	37 (57.8%)	23 (35.9%)	
Multigravida	27 (42.2%)	41 (64.1%)	

Table 2 depicts the vitamin D levels in both groups. Most patients in Group A had vitamin D levels in the range of 5-15 ng/mL (56.2%), whereas most of those in Group B had vitamin D levels in the range of 10-15 ng/mL (60.9%). With a p-value of <0.001, the difference between the two groups was deemed statistically significant.

**Table 2:** Showing Vitamin D Levels

	Group A		Group B	
<5 ng/mL	3	4.6%	1	1.5%
>5–10 ng/mL	36	56.2%	13	20.3%
>10–15 ng/mL	20	31.25%	39	60.9%
>15–20 ng/mL	5	7.8%	10	15.62%
>20 ng/mL	0	0	1	1.5%

Table 3 provides information on fetomaternal outcomes. The majority of patients in Group A delivered via C-section (75%), while most of those in Group B delivered vaginally (59.4%); the difference between the two groups was considered statistically significant, with a p-value of <0.001. Additionally, 43.8% of neonates in Group A have NICU admission, whereas 18.8% of patients in Group B have NICU admission. With a p-value of 0.007, the difference between the two groups was found to be statistically significant.

**Table 3:** Showing Mode of Delivery

Mode of Delivery	Group A	Group B	P-value
Normal Vaginal Delivery (NVD)	16 (25%)	38 (59.4%)	<0.001
Lower Segment Cesarean Section (LSCS)	48 (75%)	26 (40.6%)	
Fetal Outcome			0.007
NICU Admission	28 (43.8%)	12 (18.8%)	
Mother's Side	36 (56.2%)	51 (79.7%)	
Stillborn	0	1 (1.5%)	

## Discussion

Preeclampsia is a multisystem disorder that is a common pregnancy complication. Depending on its severity, it affects both maternal and fetal health, increasing morbidity and mortality. It is important to predict and diagnose preeclampsia early to prevent dangerous complications. To decrease morbidity and mortality related to preeclampsia, all women at risk must be screened.

This research was an 18-month facility-based observational study that aimed to determine the relationship between preeclampsia and vitamin D levels. During the study period, 128 patients were admitted to the study area’s emergency labor room. A similar methodology was used by Singla et al. [9] and Bakacak et al. [10].

In this study, the majority of women in Group A (preeclampsia) were between the ages of 18 and 23 (64.1%), whereas most women in Group B (normotensive women) were between the ages of 24 and 28 (45.3%). Moreover, the mean age in Group A was 23.8 years, while it was 25.4 years in Group B. These results were similar to those of Sajith et al. [11] and Kumari et al. [12]; in their respective papers, the mean age of preeclamptic women was 23.8 years, while the mean age of normotensive females was 26.15 years.

This paper also found a significant correlation between BMI and preeclampsia. The majority of those in Group A were overweight (25-29.9 kg/m^2^) or obese (>30 kg/m^2^), accounting for 56.2% and 17.2% of patients, respectively. In Group B, most women (75%) had a normal BMI (18.5-24.9 kg/m^2^). The mean BMI of women in Group A and Group B were 27.01 kg/m^2^ and 23.35 kg/m^2^, respectively. These outcomes are consistent with those of Sharami et al. [13], who revealed that the mean BMIs in their mild and severe preeclampsia groups were 28.99 kg/m^2^ and 26.26 kg/m^2^, respectively. Similar to ours, a study conducted by Lopez-Jaramillo et al. [14] and Bodnar et al. [15] also discovered a strong correlation between BMI and preeclampsia.

Furthermore, in this study’s Group A, there were more primigravida or nulliparous women (57.81%) than multiparous ones (42.18%). Conversely, there were more multipara (64.06%) than nullipara/primipara females (35.9%) in Group B. This indicates that nulliparity has a strong correlation with preeclampsia. In a similar study, Opitasari et al. [16] found that most women with preeclampsia were multiparous, comprising 57.5% of the sample; nulliparous females accounted for 42%. The same observation was made by Tessema et al. [17].

Moreover, this paper discovered that most women, irrespective of group, had vitamin D levels lower than normal. Notably, the vitamin D levels in preeclamptic women were significantly lower. The mean vitamin D level was 9.85 in Group A, while it was 12.58 in Group B. The majority of women in Group A had vitamin D levels in the 5-10 ng/mL range (56.2%), whereas Group B had levels in the 10-15 ng/mL range (60.9%). The difference between the two groups was statistically significant. These results coincide with those obtained by Singla et al. [9]. Similarly, Arumaikannu et al. [18] found that most women in their preeclampsia group had lower vitamin D levels; the mean vitamin D levels in their groups were 16.9 and 19.7 ng/mL, respectively. Jindal et al. [19] also found that the majority of women in their preeclamptic group were vitamin D deficient. In Fogacci et al.'s [20] study, the difference in vitamin D levels between preeclamptic and normotensive women was statistically significant. Furthermore, many studies used vitamin D supplementation as an intervention. In their meta-analysis, Fogacci et al. [20] concluded that vitamin D supplementation reduces the risk of preeclampsia. Sasan et al. [21] also conducted a similar research. Notably, both of these studies support this paper’s findings.

In this study’s Group A, 48 out of 64 women had their babies delivered through C-section, comprising 75% of the sample; Group B’s cesarean rate was only 40%. This result is similar to that of Kashanian et al. [22]. Moreover, neonates born to Group A women had a higher NICU admission rate than those born to Group B. In Group A, 43.7% of neonates were admitted, while in Group B, only 18.7% were admitted. This outcome was found to be statistically significant and also observed in studies conducted by McKenzie et al. [23] and Chapell et al. [24].

The main limitation of this study was the small sample size with the study design being an observational study. Further research (e.g., effects of vitamin D supplementation) is required to establish a relationship between vitamin D deficiency and preeclampsia.

## Conclusions

This study found that preeclamptic patients had much lower vitamin D levels than normotensive patients. Moreover, when compared to normal pregnancy, NICU admission was more common in preeclamptic patients, particularly those with low Apgar scores.

## References

[1] National High Blood Pressure Education Program Working Group on High Blood Pressure in Pregnancy (2000). Report of the national high blood pressure education program working group on high blood pressure in pregnancy. Am J Obstet Gynecol.

[2] Steegers EA, Von Dadelszen P, Duvekot JJ, Pijnenborg R (2010). Pre-eclampsia. Lancet.

[3] Sibai B, Dekker G, Kupferminc M (2005). Pre-eclampsia. Lancet.

[4] James JL, Whitley GS, Cartwright JE (2010). Pre-eclampsia: fitting together the placental, immune and cardiovascular pieces. J Pathol.

[5] Wagner LK (2004). Diagnosis and management of preeclampsia. Am Fam Physician.

[6] Cannell JJ, Hollis BW (2008). Use of vitamin D in clinical practice. Altern Med Rev.

[7] Gallagher JC, Sai AJ (2010). Vitamin D insufficiency, deficiency, and bone health. J Clin Endocrinol Metab.

[8] Adams JS, Hewison M (2008). Unexpected actions of vitamin D: new perspectives on the regulation of innate and adaptive immunity. Nat Clin Pract Endocrinol Metab.

[9] Singla M, Garg P, Sharma S (2019). Comparison of vitamin D levels in pre-eclamptic and normotensive pregnant women in a tertiary care. J Clin Diagnostic Res.

[10] Bakacak M, Serin S, Ercan O (2015). Comparison of Vitamin D levels in cases with preeclampsia, eclampsia and healthy pregnant women. Int J Clin Exp Med.

[11] Sajith M, Nimbargi V, Modi A, Sumariya R, Pawar A (2014). Incidence of pregnancy induced hypertension and prescription pattern of antihypertensive drugs in pregnancy. Int J Pharma Sci Res.

[12] Kumari N, Dash K, Singh R (2016). Relationship between maternal age and preeclampsia. IOSR J Dent Med Sci.

[13] Sharami SH, Zendehdel M, Mirblouk F, Asgharnia M, Faraji R, DalilHeirati SF, Salamat F (2017). Comparison of preeclampsia risk factors regarding to severity with control group. Zahedan J Res Med Sci.

[14] Lopez-Jaramillo P, Barajas J, Rueda-Quijano SM, Lopez-Lopez C, Felix C (2018). Obesity and preeclampsia: common pathophysiological mechanisms. Front Physiol.

[15] Bodnar LM, Ness RB, Markovic N, Roberts JM (2005). The risk of preeclampsia rises with increasing prepregnancy body mass index. Ann Epidemiol.

[16] Opitasari C, Andayasari L (2014). Parity, education level and risk for (pre-) eclampsia in selected hospitals in Jakarta. Health Sci J Indones.

[17] Tessema KF, Gebremeskel F, Getahun F, Chufamo N, Misker D (2021). Individual and obstetric risk factors of preeclampsia among singleton pregnancy in hospitals of southern Ethiopia. Int J Hypertens.

[18] Arumaikannu J, Rani SU, Shanthi S (2018). Role of vitamin D in preeclampsia: our experience. Int J Clin Obstet Gynaecol.

[19] Jindal S, Sharma JC, Sharma M (2019). Association of deficiency of maternal vitamin D levels with severity of preeclampsia. Epidemiology International.

[20] Fogacci S, Fogacci F, Banach M (2020). Vitamin D supplementation and incident preeclampsia: a systematic review and meta-analysis of randomized clinical trials. Clin Nutr.

[21] Behjat Sasan S, Zandvakili F, Soufizadeh N, Baybordi E (2017). The effects of vitamin D supplement on prevention of recurrence of preeclampsia in pregnant women with a history of preeclampsia. Obstet Gynecol Int.

[22] Kashanian M, Baradaran HR, Bahasadri S, Alimohammadi R (2011). Risk factors for pre-eclampsia: a study in Tehran, Iran. Arch Iran Med.

[23] McKenzie KA, Trotman H (2019). A retrospective study of neonatal outcome in preeclampsia at the University Hospital of the West Indies: a resource-limited setting. J Trop Pediatr.

[24] Chappell LC, Enye S, Seed P, Briley AL, Poston L, Shennan AH (2008). Adverse perinatal outcomes and risk factors for preeclampsia in women with chronic hypertension: a prospective study. Hypertension.

